# Do We Feel the Same Empathy for Loved and Hated Peers?

**DOI:** 10.1371/journal.pone.0125871

**Published:** 2015-05-29

**Authors:** Giulia Bucchioni, Thierry Lelard, Said Ahmaidi, Olivier Godefroy, Pierre Krystkowiak, Harold Mouras

**Affiliations:** 1 EA 4559, Laboratoire de Neurosciences Fonctionnelles et Pathologies, Université de Picardie Jules Verne Centre Universitaire de Recherche en Santé (CURS), CHU Sud, Nouveau Bâtiment, Rue René Laennec, F- 80054 Amiens cedex, France; 2 Service de neurologie, CHU Amiens, Place Victor Pauchet, F-80054 Amiens, France; 3 EA 3300, Adaptations Physiologiques à l’Exercice et Réadaptation à l’Effort, UFR des Sciences du Sport, Université de Picardie Jules Verne, 80025 Amiens, France; 4 Structure Fédérative de Recherche CAP-Santé (FED 4231), Pôle de Recherche et d’Enseignement Supérieur Université Fédérale Européenne Champagne Ardenne Picardie, F-51097 Reims, France; 5 EA 7273, Centre de Recherche en Psychologie: Cognition, Psychisme et Organisations, UFR de Sciences Humaines Sciences Sociales et Philosophie, Département de Psychologie, Université de Picardie Jules Verne, 80000 Amiens, France; University of Udine, ITALY

## Abstract

Empathy allows us to understand and react to other people's feelings and sensations; we can more accurately judge another person’s situation when we are aware of his/her emotions. Empathy for pain is a good working model of the behavioral and neural processes involved in empathy in general. Although the influence of perspective-taking processes (notably "Self" vs. "Other") on pain rating has been studied, the impact of the degree of familiarity with the person representing the “Other” perspective has not been previously addressed. In the present study, we asked participants to adopt four different perspectives: "Self", "Other-Most-Loved-Familiar", "Other-Most-Hated-Familiar" and "Other-Stranger". The results showed that higher pain ratings were attributed to the Other-Most-Loved-Familiar perspective than to the Self, Other-Stranger and Other-Most-Hated-Familiar perspectives. Moreover, participants were quicker to rate pain for the Other-Most-Loved-Familiar perspective and the Self-perspective than for the other two perspectives. These results for a perspective-taking task therefore more clearly define the role of familiarity in empathy for pain.

## Introduction

Empathy is a multidimensional construct with both an emotional and a cognitive component [1]: emotional empathy is the observer’s affective response to the observation, inference or imagination of another person’s feelings as a consequence of emotional sharing [2], whereas cognitive empathy is the ability to adopt another person's perspective [3], [4], [5]. Empathy therefore enables us to understand another person’s feelings by means of emotional simulation (an automatic tendency to mimic another person’s expressions) [4], [6], [7] and to cognitive processing (the ability to apply a perspective-taking process) [4], [8], [9], [10]. These bottom-up and top-down processes are activated at different times and in various situations during the empathic response and depending on the triggering situation [4], [9]. Moreover, cerebral networks are involved in the distinction between a situation involving the subject itself or another person [11]. In fact, the ability to infer another person's mental state requires the ability to inhibit the egocentric bias through which we attribute our own intentions, beliefs or emotions to third parties [12], [13], [14] and the frontopolar gyrus is a cerebral area which appears to play an important role in this inhibition [15]. Published data suggest that adopting another person's perspective and differentiating the agency of actions involve bilateral activation of the temporoparietal junction (TPJ) [16] and activation of the frontopolar gyrus for various tasks (whether motor-based [17] conceptual [15] or emotional [8], [18]).

Studies of pain perception have evidenced a degree of overlap between the neural network used to perceive one's own pain (posterior insula (PI), primary somatosensory cortex (S1) and large parts of medial and anterior cingulate cortices) and that used to imagine another person's pain (anterior insula (AI), anterior cingulate cortex (ACC), medial cingulate cortex (MCC) and thalamus) [7], [19], [20], [21]. Recently, an event-related potentials (ERPs) study [22] observed that the late, controlled component was less intense for evaluation of another person's pain (referred to in the literature as "the Other-perspective") than for evaluation of one's own pain (referred to as "the Self-perspective"). However, this was not the case for the early, automatic component of ERPs.

The working model of empathy for pain usually involves rating the level of pain felt in a visually depicted situation as rapidly as possible [7], [20], [23], [24]. Previous studies have demonstrated the influence of the link between the observer and the depicted character receiving painful stimulation on pain evaluation. For example, Pillai Riddell and Craig [25], showed differences in pain ratings between parents, nurses and pediatricians who observed a children’s reaction to a routine injection: parents rated a higher level of pain than nurses and pediatricians. Jackson et al. [20] also recorded a significantly shorter reaction time (RT) for the Self-perspective than for the Other-perspective. Pain ratings in different racial groups have been studied, but neither Xu et al. [26] nor Azevedo et al. [27] found any significant between-group differences with respect to the racial factor. However, it is well known that, at an implicit level, the emotional response and sensorimotor resonance for pain are greater for group members than for people outside the group [28]. Gender differences in empathy for pain remains a controversial issue. For example, Yang et al. [29] reported stronger mu suppression in females than in males when they looked at painful and non-painful images, but no differences in pain ratings of painful and non-painful stimuli. In another study, Han et al. [30] did not find any difference in empathy for pain ratings according to gender. However, recent studies that applied real painful stimuli reported either a higher sensitivity to painful faces [31] or higher pain rating during the observation of another person’s pain in females compared to males [32].

In a recent study [33], we demonstrated that participants tended to rate pain stimuli as more painful when they adopted an in-group member perspective rather than a self-perspective. Cheng et al. [34] asked participants to adopt three different perspectives: the participant (self), his/her partner (loved-one) or another unfamiliar person (stranger). They recorded higher pain ratings for the self and the loved-one perspectives compared to the stranger perspective, with no significant difference between self and loved-one perspectives. However, none of these behavioral studies took into account individual differences in all levels of familiarity.

In the present study, we focused on the behavioral aspect of pain evaluation and investigated the perceived intensity of pain in response to a set of previously validated pictures [7] as a function of a Self-perspective and three different Other-perspectives. We sought to determine whether the pain rating for each image varied as a function of the level of familiarity perceived by the participant. Our starting hypothesis was that the Self and Other-Most-Loved-Familiar (OMLF) perspectives would be associated with more rapid RTs and higher pain ratings than Other-Stranger (OS) and Other-Most-Hated-Familiar (OMHF) perspectives. We also analyzed the role of gender on the perceived intensity of pain in these four perspectives.

## Methods

### Participants

Sixty-six right-handed healthy participants (32 males and 34 females; mean age ± SD: 23.3 ± 5.04) took part in the study after having provided their written informed consent. Exclusion criteria were a history of visual or motor impairment and prior or ongoing treatment for psychiatric or neurological disorders. Participants were students and they received gift cards in exchange for their voluntary participation. The experiment was performed in accordance with the ethical standards of the Declaration of Helsinki and was approved by the local investigational review board (Comité de Protection des Personnes Nord-Ouest II, Amiens, France).

### Stimulus material

**Fig 1:**
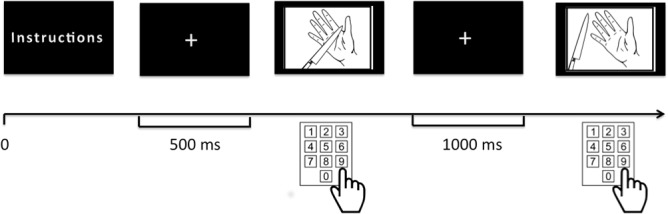
Schematic representation of the paradigm: the description of the task for each of the four blocks (Self, Other-Most-Loved-Familiar, Other-Most-Hated-Familiar and Other-Stranger) was presented and a fixation cross was displayed for 500 ms in the instruction part. An image of a painful or non-painful stimulus was presented until the participant had rated the imagined level of pain from 0 to 9 using a keyboard. After an interstimulus interval of 1000 ms, a new stimulus was displayed on the screen.

Thirty-six pictures (depicting the hand or the foot in either painful or non-painful situations) were chosen from previously validated databases [7]. When rating the pain in the picture, participants were asked to adopt the Self-perspective or one of three different Other-perspectives (OMLF, OMHF and OS). Each condition (Self, OMLF, OMHF and OS) was presented in a separated block. For each participant, the pictures were presented in random order for four times, one for each block. The four conditions were also counterbalanced across the set of pictures. Stimuli were presented using E-Prime software (version 2.0, Psychology Software Tools, Inc., Pittsburgh, PA). The experimental paradigm is depicted on Fig 1.

### Procedure

Before starting the experiment, each participant filled out a series of standardized questionnaires, including the French version of the Beck Depression Inventory (BDI) [35], the French version of State-Trait Anxiety Inventory, the subscales T (trait) and E (state) [36] and the French version of the 20-item Basic Empathy Scale (BES) [37,38], with 9 and 11 items assessing cognitive empathy and affective empathy, respectively. The BDI II and STAI were used to exclude any participants with psychiatric disorders such as moderate or severe depression and elevated levels of anxiety. A score higher than 19 on the BDI constituted an exclusion criteria (cut-off score of 20 for a diagnosis of mild depression). For the T and E subscales of the STAI questionnaire, subjects with scores more than two standard deviations higher than the median French population score were excluded. Considering these criteria, two participants were excluded from this sample. The BES was used to check the level of the participant’s empathy and establish correlations with the participant’s responses. We chose to administer the BES because it provides better evaluation of cognitive empathy than the Interpersonal Reactivity Index [37], [39]. Behavioral variables (pain rating and reaction time, i.e. time lag between presentation of the picture and pain rating; RT) were recorded using E-Prime software (version 2.0, Psychology Software Tools, Inc.). The pain-rating task was organized as follows: at the beginning of each block (with one block for each familiarity condition), participants were instructed to imagine the pain that they (Self) or someone else (OMLF, OMHF and OS) would experience in each situation presented. More specifically, the following instructions were provided at the beginning of each block: “Dear participant, you are going to see a series of images presented on the computer screen. We would like you to rate the intensity of pain that you (in the SELF block) / the most loved familiar person (in the OMLF block) / the most hated familiar person (in the OMHF block) / someone you don’t know (in the OS block) would experience in the situation displayed; please indicate your rating by selecting a number between 0 (no pain) and 9 (worst possible pain) on the keyboard using your right hand”. We masked the purpose of our study, by not using the word ‘empathy’ in the instructions, although we did not check whether participants were aware of the purpose of the study. The order of presentation of the four blocks (Self, OMLF, OMHF and OS) was counterbalanced. Each block started with the presentation of a fixation cross for 500 ms. The stimulus was then presented until the participant responded. After the response, an interstimulus interval of 1000 ms was added. The participant’s task consisted in replying as rapidly as possible after presentation onset. The participant indicated the rating on a keyboard with his/her right hand. Pain ratings were recorded on a 10-point Likert scale between 0 (no pain) and 9 (worst pain imaginable). Persons designated as "most loved" (OMLF) and "most hated" (OMHF) were also divided into four categories: (i) parents and other relatives, (ii) friends and acquaintances, (iii) partners and (iv) teachers and bosses.

### Statistical analysis

All data were analyzed using the Statistical Package for the Social Sciences (version 21.0, SPSS Inc., Chicago, IL, USA). Trials with scores 2.5 SD above or below each individual mean for each condition in the pain rating task were excluded as outliers (1%). Two separate repeated-measures analyses of variance (ANOVAs) were performed on pain ratings and RTs, with gender as a between-subjects factor. Another repeated-measures ANOVA was then performed on pain rating data, with perspective (Self, OMLF, OMHF, OS) and stimulus value (painful, not painful) as within-subject factors and gender as a between-subjects factor. As the RT values were not normally distributed, they were log-transformed prior to the ANOVA. Paired-sample t-tests with a Bonferroni correction were used to compare the pain ratings for painful stimuli. Pearson’s correlation coefficients were also calculated for the relationship between the participants’ pain ratings for painful images and their level of empathy on the BES. The limit for statistical significance was p<0.05 for all statistical analyses.

## Results

**Fig 2:**
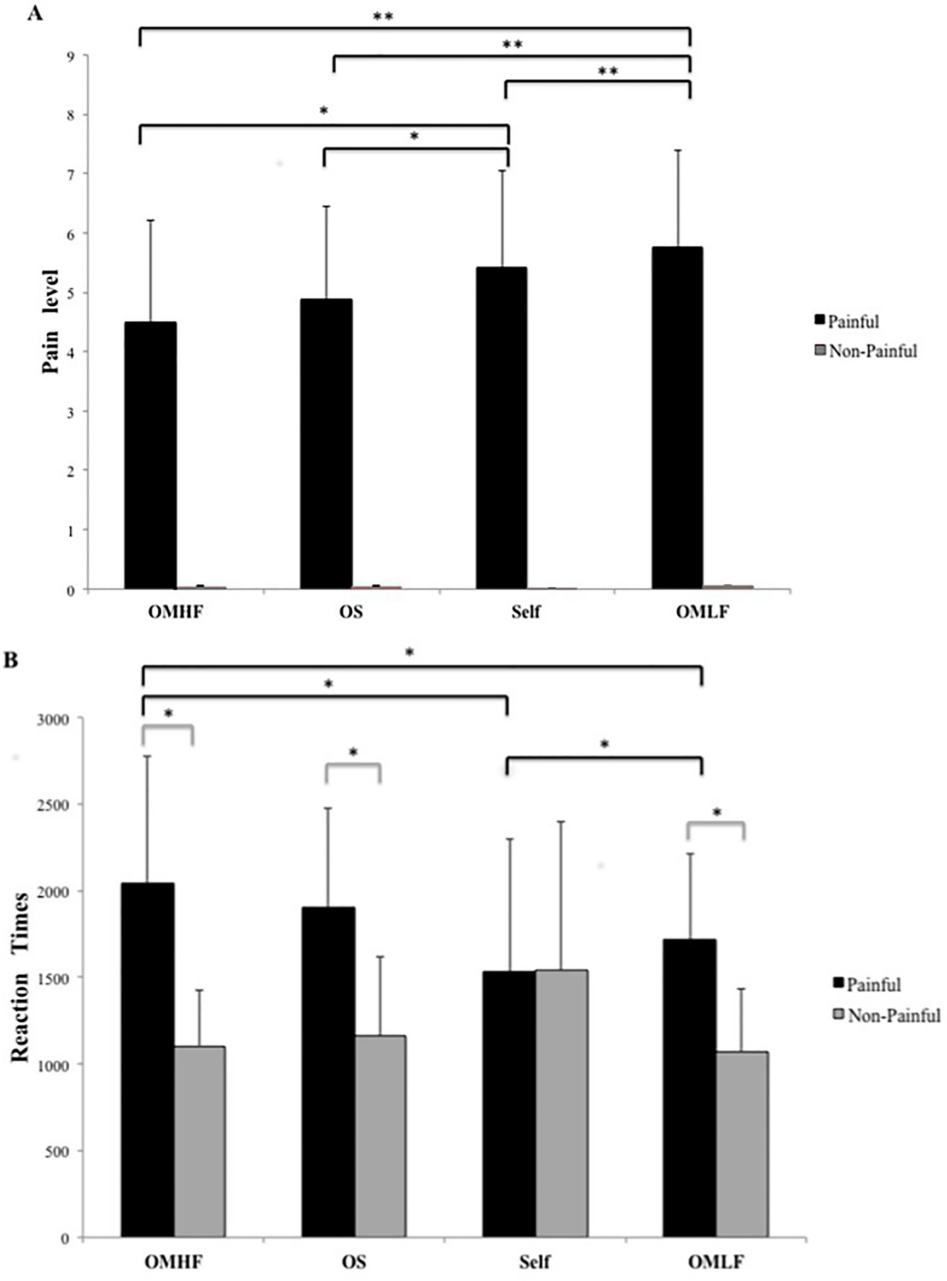
(A) Mean ± SD pain ratings as a function of the stimulus (painful vs. non-painful) and the perspective (Self, OMLF, OMHF and OS). Significant differences are indicated as: *p < 0.05 **p < 0.001 (B) Mean ± SD RTs as a function of the stimulus and the perspective.

Behavioral results are reported in Tables 1 (pain ratings) and 2 (reaction times) (see also Fig 2 (A) and (B)). For the OMLF perspective, 41% of the designated people were parents or relatives, 59% were partners, and none were friends, teachers or bosses. For the OMHF perspective, 1.7% of the designated people were parents or relatives, 88.1% were friends or acquaintances, 10.2% were teachers or bosses, and none were partners.

**Table 1:** Pain ratings (Mean values ± SD) as a function of stimuli (painful vs. non-painful) and perspective (other-negative familiar, other-unknown, self, other-positive familiar).

	OMHF	OS	Self	OMLF
**Painful**	4.50 ± 1.71	4.89 ± 1.55	5.42 ± 1.63	5.76 ± 1.63
**Non-Painful**	0.04 ± 0.21	0.05 ± 0.20	0.02 ± 0.04	0.06 ± 0.23

**Table 2:** Response time (Means values ± SD) as a function of stimuli and perspective.

	OMHF	OS	Self	OMLF
**Painful**	2044.4 ± 731.2	1106.2 ± 568.7	1534.1 ± 766.7	1719.2 ± 493.5
**Non-Painful**	1106.2 ± 316.6	1160.6 ± 456.4	1544.3 ± 851.3	1074.1 ± 357.4

### Pain ratings

An ANOVA with respect to the perspective condition (Self, OMLF, OMHF, OS) revealed a significant main effect [F(3,65) = 19.51; p<0.0001]. The main effect of stimulus value (painful or non-painful) was also statistically significant [F(1,65) = 897.19; p<0.0001]. A significant interaction was demonstrated between the perspective and the stimulus condition [F(3,65) = 20.65; p<0.0001]. The interactions between perspective and gender [F(3,65) = 4.50; p<0.005], valence and perspective [F(3,65) = 4.23; p<0.05], and valence and gender [F(3,65) = 4.11; p<0.05] were also statistically significant.

*Post hoc* pairwise comparisons were finalized with *t*-tests and the Bonferroni correction was applied. Mean pain ratings were higher in the OMLF perspective compared to the OMHF perspective (t65 = 6.06; p<0.0001), OS perspective (t65 = 6.36; p<0.0001) and Self perspective (t65 = 4.12; p<0.0001) (see Fig 2 panel A). The mean pain ratings (Table 1) were significantly higher for the Self-perspective than for the OS (t65 = 3.03, p<0.005) and OMHF (t65 = 3.89; p<0.0001) perspectives. Although pain ratings were higher for the OS perspective than for the OMHF perspective, the difference was not statistically significant. As a between-subjects variable, gender had a significant effect [F(1,65) = 4.20; p<0.05]. Female participants rated the painful stimuli as more painful in the OMLF (t65 = 3.38; p<0.005) and Self (t65 = 2.98; p<0.005) conditions than male participants. A t-test was also used to compare the Other-perspectives as a whole (i.e. the pooled OMLF, OMHF and OS data) with the Self-perspectives. Painful images were rated as significantly more painful for a Self-perspective than for an Other-perspective (t65 = 2.29; p<0.05).

### Reaction times

Mean and standard deviations of RTs in the four conditions (Self, OMLF, OMHF, OS) in painful and non-painful situations) are reported in Table 2. The ANOVA of the log-transformed RT data revealed a significant main effect of perspective condition [F(3,65) = 4.76; p<0.005] and stimulus value [F(1,65) = 298.89; p<0.0001]. A significant interaction was demonstrated between perspective and stimulus value [F(3,65) = 64.95; p<0.0001]. No gender effect on RT was observed on t-tests with Bonferroni correction. The mean RT for painful stimuli was shorter in the OMLF condition than in the OS condition (t65 = -3.43; p<0.001) and OMHF condition (t65 = -5.37; p<0.0001), but was longer than in the Self-condition (t65 = 4.04; p<0.0001) (see Fig 2, panel B). The RTs were significantly longer for the OMHF perspective than for the Self-perspective (t65 = 7.80; p<0.0001). No significant differences in RTs were observed between females and males.

### Correlations

None of the correlations between pain ratings and BES scores (whether total, cognitive or emotional) were statistically significant (for example, r = 0.212 for the correlation between mean total BES score and the pain rating for painful situations with the Self-perspective, and r = 0.234 for the correlation between mean total BES score and the pain rating for painful situations with the OMLF-perspective). The RTs recorded for painful stimuli with the OMHF perspective were positively correlated with the total BES score (r = 0.25; p = 0.05). The RTs recorded for painful stimuli with the OMLF perspective were positively correlated with the BES cognitive subscore (r = 0.29; p = 0.05). The BES cognitive subscore was also positively correlated with the difference in RTs between painful and non-painful stimuli with an OMLF perspective (r = 0.28; p = 0.05).

## Discussion

Empathy enables us to understand and share another person's feelings. As such, empathy plays an essential role in social interactions between humans [40]. Perception of another person in a painful situation involves much of the neural network activated during first-person experience of pain (for a meta-analysis, see Lamm et al., 2011 [21]).

In the present study, participants were asked to rate the level of pain felt from their own perspective or that of other people. Our results are in agreement with data published in the literature: the participants rated painful images as more painful when adopting a Self-perspective than when adopting an Other-perspective [20]. Moreover, our experimental design distinguished between three levels of familiarity within the Other perspective: the OMLF, OMHF and OS perspectives. The situations were rated as most painful when the participants adopted an OMLF perspective (usually that of their mother or their partner). This differentiation provides a finer judgment of the degree of painfulness attributed to each context: a painful situation is considered to be most painful when it is experienced by a loved one, then by ourselves, then by a stranger and, lastly, by someone we hate (usually a former partner or friend, or a current teacher or boss). These results are in agreement with data published in the literature [7], [20], [22] showing that painful stimuli are rated as more painful with a Self-perspective than with an Other-perspective. However, none of the previous studies focused on the level of familiarity with the "Other". Our findings remained the same when data for the Other-perspectives were pooled; the mean pain rating was significantly lower than for a Self-perspective (in line with the literature).

If we assume that the OMLF perspective is similar to an "in-group" perspective, the present results are in agreement with our previous report [33] of significantly higher pain ratings for an in-group perspective than for Self- and out-of-group perspectives. In agreement with some previous studies [20], [22] but not others [33], we found that adoption of a Self-perspective was associated with shorter pain rating RTs than Other-perspectives (OMLF, OS or OMHF). The absence of this finding in the study by Montalan et al. (2012) [33] may have been due to their smaller sample size. Furthermore, we found that the OMLF perspective was associated with the second shortest mean RTs, suggesting that the in-group perspective [33] may have been more complicated to adopt, since there was no difference in RTs between the in-group and out-of-group perspectives. Pain ratings were higher for the Self-perspective than for the Other-perspectives because the Self-perspective is more closely related to the participant’s own, real experience of pain (as reported in [20]). This finding was confirmed by the shorter mean RTs: direct experience of pain would enable more rapid rating of painful or non-painful situations. Rating pain from an Other-perspective requires more time.

Pain ratings for the OMLF perspective were even higher than for the Self- perspective. This finding confirms the hypothesis in which the level of empathy depends on affective proximity. It also agrees with previous work showing that participants produce more intense empathic responses when considering a familiar person (such as a parent, child [25] or loved one [34]) than when considering a stranger. According to Aron et al. [41], [42], the closer the relationship between two individuals, the more they are integrated into a Self-perspective. Indeed, the degree of familiarity between two people may influence the degree of empathy felt for another person [43]. Moreover, factors such as altruism (i.e.: helping others without thinking about your direct advantage) may also influence the level of empathy felt by study participants. Batson [44] proposed the empathy–altruism hypothesis, in which empathy helps to improve another person's well-being [44], [45], [46]. As previously suggested [33], we consider that this overlap between mental representations of Self and Other might explain the higher mean pain ratings for the OMLF perspective. Furthermore, our observation of significantly lower mean pain ratings for the OS and OMHF perspectives also fits with this hypothesis. The absence of a significance difference between the OS and OMHF perspectives may have been due the participants' unfamiliarity with adopting these two perspectives and the sometimes small perceived difference between them to bias. Moreover, it is noteworthy that the longest mean RTs for pain ratings were recorded for the OMHF perspective. The presence of inhibition during pain ratings for this perspective can be legitimately hypothesized.

Although previous studies conducted according to a similar methodology [29], [30] have failed to highlight a significant difference between genders in pain ratings in an empathy for pain task, pain stimulation studies [31], [32] demonstrated gender differences in rating the level of another person’s pain. The influence of gender on empathy for pain remains unclear at the present time. We analyzed whether the gender variable could modulate empathy for pain ratings using self / different other perspectives. In particular, the results suggest that female subjects are more sensitive than males in rating their pain and the pain of their loved ones in line with pain stimulation studies [31], [32]. There are several examples in the literature supporting the superior empathy capacity of females [47], but they are often based on self-report questionnaires [48], as in the present study. For these reasons, these results must be interpreted cautiously and need to be reproduced by further studies.

RTs recorded during the OMHF condition were positively correlated with the total BES score: participants who spent more time rating painful conditions during the OMHF perspective probably had a higher level of empathy than other participants. Moreover, both the painful condition and the delta between painful and non-painful conditions with the OMLF perspective were positively correlated with the cognitive BES subscale, that assesses the intellectual understanding of another person’s mental state [38]. Cognitive empathy is based on important cognitive functions such as executive functions and language [40]. It is important to more clearly understand the loved person’s feelings, especially when the imagined person is thought to be in a dangerous situation (i.e. when the subject has to determine the level of pain that the imagined loved person would experience). Perspective-taking requires a lot of energy to maintain the interaction between limbic and high-level cognitive structures and a greater volume of information needs to be processed after a certain period of time [49]. This process then becomes less selective and slower, as it becomes painful to imagine the loved person in pain, which is probably why subjects with a higher level of empathy tend to devote more time to this task.

Zeki and Romaya [50] showed that the neural correlates of hate involve the premotor cortex, a region involved in motor planning. The authors hypothesized that seeing a hated person activates the premotor system to apply approaching / avoidance behavior. There is also evidence in favor of the existence of freezing behavior during observation of another person’s pain that is specific to the muscle vicariously involved in the painful stimulation [51]. We can also hypothesize that motor response RT freezes more severely when the subject has a higher level of empathy towards his/her hated peers. Cognitive hypothesis must also be taken into account. It can be assumed that this task required more cognitive resources in relation to the OMLF condition. Subjects may feel inhibited to attribute higher pain ratings in the OMHF condition compared to the OMLF condition, which would account for the longer reaction times [52].

In conclusion, these findings confirm the hypothesis that the level of empathy depends on affective proximity [25,34]. Moreover, this study completed and clarified the behavioral results of previous studies [20], [22], [34], separating the “Other” perspective into three different levels of familiarity and analyzing the differences in pain perception with respect to these perspectives. This study showed that imagining the most loved person in a painful situation is associated with higher pain ratings and lower reactions times. On the contrary, imagining the most hated person in a painful situation is associated with lower pain ratings and higher reactions times. Overall, these results suggest that the type of relationship between the participant and the observed person in pain may modulate the way in which interpersonal factors influence pain perception.

## Supporting Information

S1 File Table of the posturographic data in the three experimental conditions. (XLSX)
